# Scalable Engineering of 3D Printing Filaments Derived from Recycling of Plastic Drinking Water Bottle and Glass Waste

**DOI:** 10.3390/polym16223195

**Published:** 2024-11-17

**Authors:** Arafat Toghan, Omar K. Alduaij, Moustafa M. S. Sanad, Noha A. Elessawy

**Affiliations:** 1 Chemistry Department, College of Science, Imam Mohammad Ibn Saud Islamic University (IMSIU), Riyadh 11623, Saudi Arabia; 2 Chemistry Department, Faculty of Science, South Valley University, Qena 83523, Egypt; 3 Central Metallurgical Research and Development Institute, (CMRDI), P.O. Box 87, Helwan, Cairo 11421, Egypt; 4 Computer Based Engineering Applications Department, Informatics Research Institute, Alexandria 21934, Egypt

**Keywords:** solid waste management, recycling, glass waste, polyethylene terephthalate (PET), 3D printing filament composite, coupling agent

## Abstract

The most significant challenge that the world is currently facing is the development of beneficial industrial applications for solid waste. A novel strategy was implemented to produce a composite with varying loadings of glass waste nanoparticles (GWNP) in 5, 10, and 15 wt.% with recycled polyethylene terephthalate drinking water bottle waste (RPET). This strategy was based on glass and drinking water bottle waste. An analysis was conducted to evaluate the performance of the composite as filaments for 3D printer applications. This study evaluated the effect of GWNP addition on the chemical structure, thermal and mechanical characteristics of the composite. The Fourier Transform Infrared (FTIR) spectra of the filament composites and RPET composites exhibited similarities. However, the mechanical strength and thermal stability of the filament composites were enhanced due to the increased GWNP content. Furthermore, the results indicated that the filament developed could be utilized for 3D printing, as demonstrated by the successful fabrication of the filament composite, including 5 wt.% GWNP, using a 3D printer pen. The production of filaments using GWNP and RPET matrix presents a cost-effective, high-yield, and ecologically beneficial alternative. The present study may pave the way for the future advancement and utilization of 3D printing filaments by treating hazardous waste and using more ecologically friendly materials in design applications.

## 1. Introduction

Rapid progress in sustainable materials technology has encouraged the investigation and development of alternative, ecologically suitable materials for different uses, including filaments of 3D printing. Due to its multifunctionality, 3D printing has emerged as an essential tool for innovative enterprise solutions. In addition, 3D printing offers fast prototyping, waste reduction, large-scale customization, and enhanced design flexibility in several industries [1]. Prototyping has become more economical across a range of industries, including the production of complex 3D tools [2].

Polyethylene terephthalate (PET) is the predominant thermoplastic type commonly available on the market. In addition, it is highly valuable due to its outstanding properties such as chemical resistance, electrical, mechanical, and thermal properties. PET finds application in the manufacture of food packaging, Tupperware, bottles for water drinking, and waterproofing. The global use of plastic bottles has resulted in a rise in the municipal solid waste streams and environmental issues. Although bulks of waste are disposed of in landfills, only a small percentage is recycled [3]. Recycling PET water bottles waste has significant environmental advantages as it effectively decreases waste, energy consumption, resource usage, and greenhouse gas emissions and improves the quality of the environment sources such as air and water [4]. However, the most extensively utilized materials for 3D printing in the fused filament fabrication (FFF) and fused deposition modeling (FDM) styles is PET due to its outstanding recycling potential, mechanical properties, and advantageous melting temperature of 260 °C [5,6,7]. Furthermore, PET-based filament is widely marketed as a highly colorable, translucent filament and produces minimal odor during the 3D printing procedure. A previous study [6] revealed that a 3D-printed PET filament, which had undergone five recycling cycles, retained its mechanical properties throughout its lifespan. Nevertheless, there was a 10 percent decrease in elongation at the fracture location. Therefore, PET waste has been demonstrated to be a more sustainable and reliable alternative to real wood [8]. In addition to its non-toxicity and high recyclability, it provides financial advantages to both the company and its customers. Utilizing recycled PET (RPET) provides a cost-effective and environmentally beneficial substitute for conventional materials like wood [9,10]. RPET is a suitable material for 3D printing because of its improved melting point, melt flow index, glass transition temperature, thermal expansion coefficient, tensile strength, and affordable price [11]. Sustainability of the environment is yet another important application of RPET.

Reinforcing materials, such as ceramics [12,13] or glass particles [14], are less effective at strengthening than fiber-reinforced composites. However, they improve stiffness, strength, and toughness. Particles can also be used as reinforcement materials due to their cost-effectiveness, ease of production, and ease of form. The utilization of glass waste as a reinforcing material in polymer matrices offers several benefits, mainly its abundance, low cost, and thermal stability. The incompatibility of thermoplastic polymer with glass powder is one of the challenges to the production of thermoplastic glass powder composites. The use of a coupling agent that reacts with the polymer matrix and glass powder is the key to enhancing the compatibility between both of these components. However, maleated polyolefins are the most commonly used coupling agents [15,16]. Conversely, when the preparation temperature is reached, the maleated polyolefins have the ability to establish multiple chemical bonds with the hydroxyl groups present on the surface of the glass particles. This interaction firmly bonds the polymeric chain to the glass particles [16].

This research aims to develop a “Green 3D filament” that can be accomplished by incorporating a specific quantity of glass waste nano-powder (GWNP), up to 15% wt.%, into recycled drinking water bottle waste (RPET) matrix in the presence of malic acid as a coupling agent. In addition, using a simple and reproducible method, this work addresses multi-scale and multi-functional challenges such as little heating power required for upcycling glass waste, contributing to sustainable waste materials processing. The thermal stability and mechanical properties of the RPET/GWNP composite were examined. The preliminary results of the study indicate that these filaments have the potential to serve as eco-friendly 3D printing filaments, which are distinguished by their distinctive aesthetic appearance and promising properties. Moreover, this method of eco-construction generates novel opportunities and promotes sustainability by combining recycled materials.

## 2. Materials and Methods

### 2.1. Fabrication of the Filament

**Figure 1:**
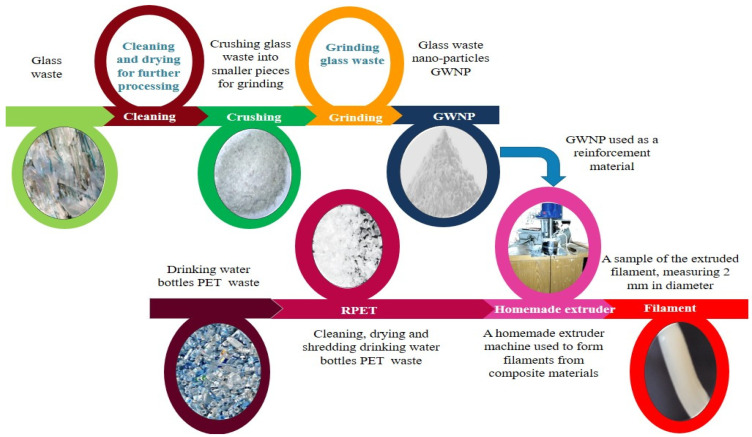
Schematic illustration of the procedure followed to prepare and test RPET/GWNP composite filament.

The production of filaments involves two primary steps, as demonstrated in Figure 1. The first step involved the preparation of glass waste to be nanosized using a “top-down” approach [17]. Initially, a waste plate glass was used and it underwent a cleaning process to eliminate dust and debris. Afterward, it was manually crushed and dried in an electrical oven overnight at 80 °C. Finally, the shredded, dried particles were ground into NP particles to produce GWNP by using a ball mill (Fritsch Pulverisette 7, Idar-Oberstein, Germany) at 600 rpm for 60 min with a mill cup capacity of 250 mL containing zirconia balls (the ball-to-powder weight ratio was 20:1). The second step involved an extradition and composition mixture of PET waste from the drinking water bottles. Afterward, it was cut into the corresponding (1–3 mm) size fractions. After that, it was mixed with GWNP in several ratios (5, 10, and 15 wt.%) and coupling agent (malic acid, Sigma-Aldrich) in different ratios (0.5, 1, and 1.5 wt.%). Subsequently, the mixture was subjected to extrusion at 270 °C in a handmade extruder to create 2 mm filament.

### 2.2. Characterizations

The GWNP was verified using a scan electron microscope (SEM, JEOL Jsm 6360LA-Tokyo, Japan) and Better Size Instruments Ltd., China. In a nitrogen environment, thermogravimetric analysis was conducted on both RPET and RPET/GWNP nanocomposites. The measuring device employed was the SDT 650 (TA Instruments, New Castle, DE, USA), with a recorded heating rate of 10 °C min^−1^, with a maximum temperature range of 30 to 800 °C. The melt flow rate (MFR) was determined using a DHR/2 (Discovery-Hybrid-Rotational-Rheometer, TA-Instruments, New Castle, DE, USA). The chemical structures of the filaments were examined using surface functional group analysis using Fourier Transform Infrared (ALPHA II-Bruker, Karlsruhe, Germany) as the transmittance mode. Tensile tests were conducted on the filament with a diameter of 2 mm. Tensile tests were performed at room temperature and 5 mm/min elongation speed on matching specimens utilizing Shimadzu Autograph 5 KN, Japan. Three specimens from each filament were selected for testing.

### 2.3. Printing 3D Pen

Using a 3D printing pen, the RPET/GWNP filaments were produced in order to concept-proof the filaments. The 3Doodler pro+ pen was acquired from Wobble Works, Inc. in the United States. It is designed to operate more efficiently than any other 3D pen due to its robust dual-drive system. In order to achieve the desired flow rate, 10 mm^3^ sec^−1^, and melting point, the printing temperature was increased to 270 °C and the pen was equipped with a 2 mm nozzle.

## 3. Results and Discussion

### 3.1. Characterization of GWNP and Filaments

**Figure 2:**
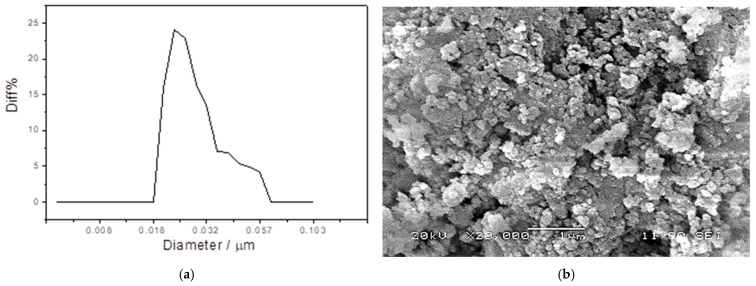
Particle size distribution (**a**), and SEM micrograph (**b**) of GWNP sample.

The size of the particles that were produced after the grinding of the glass was examined. The nanosized scale was verified using Better Size Instruments Ltd.’s, Dandong, China particle size distribution as demonstrated in (Figure 2a,b). The average particle diameter of GWNP ranged from 20 to 60 nm.

**Figure 3:**
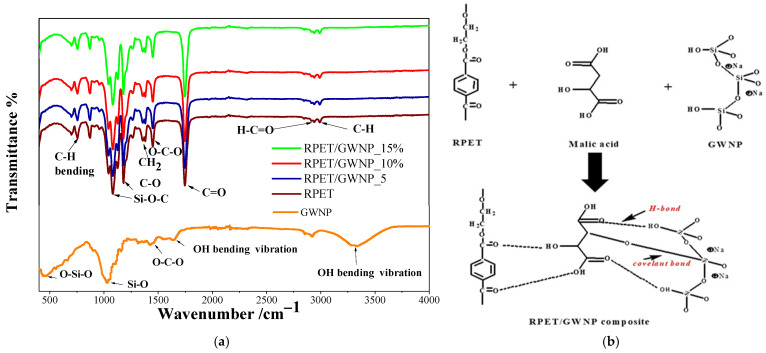
(**a**) FTIR spectra, (**b**) suggested structure for RPET/GWNP composite filament.

The FTIR spectra of the composite filaments and raw materials are displayed in Figure 3a. GWNP was present in varying quantities in the three composite filaments that were produced: RPET/GWNP_5%, RPET/GWNP_10%, and RPET/GWNP_15%. Multiple peaks are noticed at various wavenumbers, and their intensities fluctuate with increases of the GWNP weight ratio from 5% to 15%. Aliphatic structures are also responsible for the peaks at 2988 and 2905 cm^−1^, which are attributed to ethyl groups and the C-H stretching of carboxylic, respectively, in addition to CH_2_ bending and CH_2_ rocking at 1387 and 874 cm^−1^ [18]. In contrast, the carboxylic ester group’s distinctive peak for C=O stretching is located at 1712 cm^−1^. The PET identification peaks, ranging from approximately 1500 cm*^−^*^1^ to 500 cm*^−^*^1^, are known to have a very complex series of absorptions [19]. For instance, the aromatic ring’s out-of-plane C–H bending is represented at 730 cm*^−^*^1^ [20].

Nevertheless, the absorption bands of the RPET/GWNP composite exhibit a certain resemblance to those of RPET, and their intensity rises as the GWNP weight ratio rises from 5% to 15%. Furthermore, it was observed that the Si–O–Si band of GWNP in the range of 1050–1150 cm^−1^ was replaced by the Si–O–C band at 800–1150 cm^−1^ in the composite filaments. The width and intensity of this band increased with higher GWNP concentration, indicating a change in the surface group of silica to Si–O–C from Si–OH and the chemical bonding of a coupling agent to the RPET surface and GWNP, as shown in Figure 3b.

**Figure 4:**
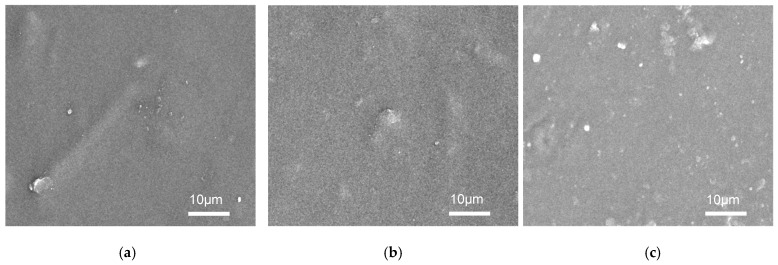
SEM micrographs for (**a**) RPET/GWNP_5%, (**b**) RPET/GWNP_10%, and (**c**) RPET/GWNP_15%.

A surface morphological analysis of RPET/GWNP composite filament was conducted using SEM, as depicted in Figure 4. Compared to the various concentrations of the reinforced GWNP, it was observed that in line with other research, the size of the aggregation grew as the weight ratio in the polymeric matrix increased [21].

**Figure 5:**
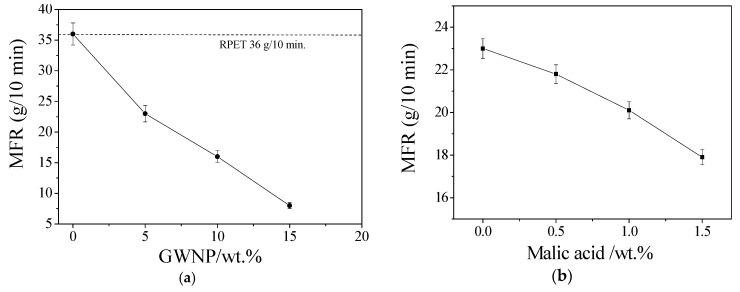
(**a**) Melt flow rate vs. GWNP (wt.%) for RPET and RPET/GWNP composites, (**b**) melt flow rate vs. coupling agent (wt.%) for RPET/GWNP-5%.

The melting flow ratio (MFR) of RPET and RPET/GWNP composites is displayed in gm/10 min in Figure 5a, and the impact of coupling agent addition on the MFR is examined in Figure 5b. Although the MFR decreases as the GWNP and coupling agent weight ratio increase, shearing resistance is typically increased as the coupling agent weight ratio rises [22]. In the instance of RPET, the maximum MFR was determined. This was anticipated due to the fact that GWNP and malic acid reduce the flow of polymers and increase the viscosity of composites. As a result, the composite filament was prepared for further research by applying a coupling agent with a weight ratio of 0.5 wt.%.

**Figure 6:**
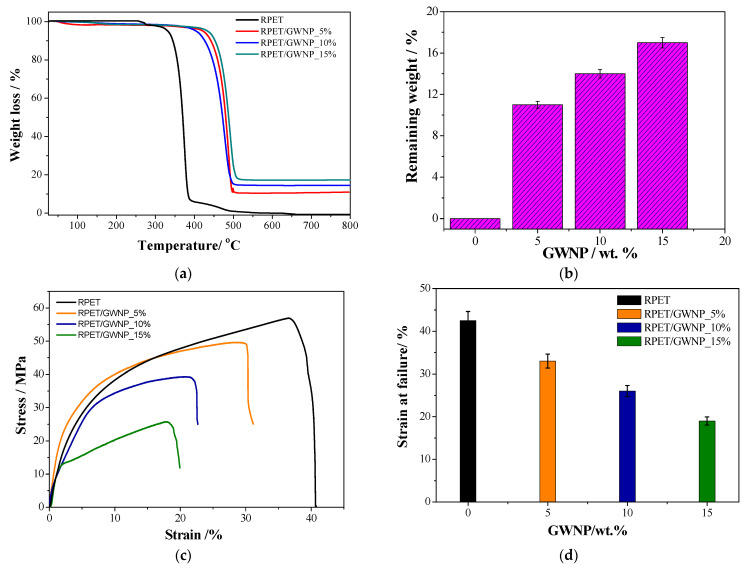
(**a**) TGA spectra for RPET and RPET/GWNP composites, (**b**) the relation between the remaining weight % vs. GWNP wt.%, (**c**) stress–strain behavior of RPET and RPET/GWNP composites obtained from the tensile test, (**d**) the relation between strain at failure vs. GWNP (wt.%).

Figure 6a demonstrates the RPET and RPET/GWNP composites thermal stability at temperatures ranging from 25 °C to 800 °C with a minimum coupling agent concentration of 0.5 wt.% and varying GWNP loading percentages. The primary big-stage degradation process across a temperature range of 300–400 °C resulted in the loss of RPET mass. This stage was preceded by a minor dehydration stage at 253 °C, PET’s melting point [23]. Furthermore, a slight degree of degradation was seen within the 400–500 °C temperature range. Conversely, the thermal degradation of RPET/GWNP composites occurred abruptly between 400 and 550 °C, and there were no mass changes throughout the entire 400 °C temperature range. This shift was exacerbated by elevating the GWNP wt.% from 5% to 15% in RPET/GWNP composites, which exhibited a slight red shift in the first degradation temperature. The volatilization of the composites is reduced, and their thermal stability is increased by the addition of more filler as GWNP does not degrade under the experimental conditions and instead remains as a residue [24,25]. The new composition offers several advantages for objects printed using Fused Filament Fabrication (FFF) and Fused Deposition Modeling (FDM) techniques. Since PET is a highly stable base material, it provides an environmentally friendly alternative capable of enduring damage, deterioration, and other typical issues encountered by conventional materials. Consequently, the utilization of RPET in 3D printing materials emphasizes its significance for ecologically sustainable applications. Additionally, it indicates that it has the potential for wider uses in other 3D printing applications.

The weight loss decreases as the GWNP wt.% increases, as shown in Figure 6b. In addition, the residual mass verified that each composite was loaded with GWNP. As hypothesized, the addition of GWNP marginally increases the temperature at which the degradation occurs. Moreover, the temperature at which the composites are 3D printed is roughly 270 °C, significantly less than the temperature at which the composites start to deteriorate sharply. This ensured that the experiment’s RPET/GWNP filament composite did not degrade, which would have affected the outcomes.

Stress–strain graphs for RPET and RPET/GWNP composites containing 5–15 wt.% GWNP are displayed in Figure 6c. The GWNP weight fraction raises the strength and modulus. Figure 6d demonstrates that RPET/GWNP_15% provides the lowest strain at failure, which is less than half of that of RPET. Subsequently, RPET/GWNP_10% exhibits a strain at a failure value of 64% lower than that of RPET. Furthermore, as the GWNP content increased, the material stiffness increased, indicating that the molecules were less mobile due to an inadequate particle size distribution [9]. However, the decline in tensile stress and modules at higher GWNP concentrations can be attributed to the diminished stress transfer area resulting from increased GWNP loading. As a result, the nanocomposite exhibits increased brittleness and reduced tensile strength when the weight percentages of GWNP are high, primarily due to a decrease in stress transfer area. This tensile modulus action for RPET reinforced with waste glass fibers and waste marble dust composites has previously been reported [24,26]. In order to determine the optimal design features for 3D printed products, the results that were gathered were considered. These outcomes will also be used as selection criteria for forms made using generative design software. The RPET/GWNP_5% filament composite was determined to be the optimal 3D printing filament in light of the aforementioned findings. It was synthesized from RPET and GWNP, with 0.5 wt.% malic acid added as a coupling agent.

### 3.2. Optimizing the Filament Content to Enhance the Mechanical Properties

A central composite design (CCD) statistical method was used to optimize the filament content to improve mechanical characteristics. The CCD approach enables the comprehensive investigation of a response surface by assessing the impacts of several independent variables [27]. For optimization, the study examined two independent variables: the weight percentage of GWNP (variable coded A, which ranged from 5 to 15 wt.%) and the weight percentage of the coupling agent (malic acid) (variable coded B, which ranged from 0.5 to 1.5 wt.%). Table 1 illustrates the design of 13 experimental runs for the CCD, which included five focal points to evaluate the reproducibility of the results. The statistical program Design-Expert v13.0 from Stat-Ease was used to process the acquired data.

An analysis of variance (ANOVA) in one direction was used to determine how significant the results were. The statistical significance was set at a *p*-value threshold of <0.05. The statistical relationship between variables and responses, specifically tensile stress (Y), was modeled using linear dependence based on the following equation:

Y = 38.77 − 14.62A − 8.09B(1)

**Figure 7:**
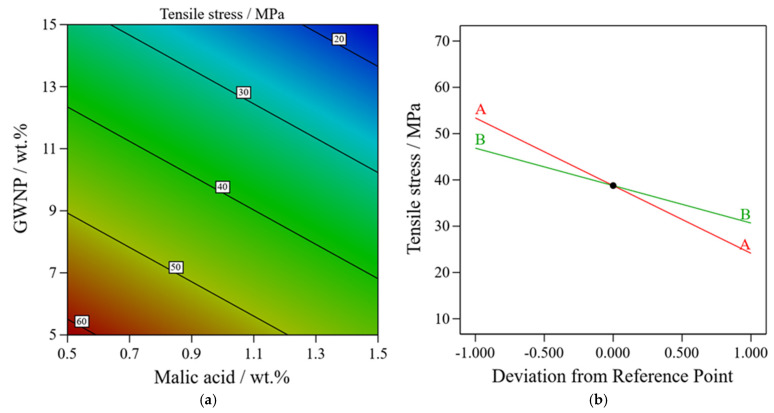
(**a**) Contour plots, (**b**) perturbation plot of GWNP (coded A) and malic acid (coded B) wt.% effect on tensile stress of the filament composite.

There is a significant correlation between the model and the experimental data, as indicated by the model’s coefficient of determination (R^2^) of 0.86. Analysis of variance (ANOVA) was used to evaluate the model’s performance and validity in further detail, as indicated in Table 2. According to the findings, the model appears to be statistically significant (*p* < 0.0001) and can accurately predict the tensile stress values using GWNP and malic acid wt.%. The model’s significance is also substantiated by the Model F-value of 30.71. It seems doubtful that noise or random variation contributed to the model’s performance when the F-value is this high. A strong correlation between the tensile stress and the GWNP and malic acid wt.% is further indicated by the adjusted R^2^ coefficient of 0.8320 and the predicted R^2^ of 0.7117. The model appears to have strong predictive power, as evidenced by the reasonably close agreement between the adjusted R^2^ and anticipated R^2^. The model’s predictions and the measured tensile stress levels are in good agreement, as seen by the little discrepancy (less than 0.2) between these two values. The filament’s tensile stress was most negatively impacted by the GWNP weight percentage, as demonstrated in Figure 7. This suggests that increasing the GWNP content reduced the composite’s tensile stress, followed by malic acid wt.%.

According to the liner model, the optimal conditions for the filament composite were determined to be 5 wt.% of GWNP, and 0.5 wt.% of malic acid should be added to RPET to achieve the maximum tensile stress of 61.485 MPa. The optimum ratio of malic acid (0.5 wt.%) was used to fabricate the tested filament composites with the printing pen.

### 3.3. 3D Printing Test

One obvious benefit of 3D printing over traditional production techniques is its ability to create designs with more complicated shapes [28], particularly during the prototyping phase. The dynamic needs align nicely with the capacity to print complex designs on demand [29]. By reducing waste formation through precise manufacturing techniques and incorporating unique natural materials in the production of filaments, 3D printing technology contributes to the growth of eco-friendly design techniques. However, the use of RPET and GWNP in 3D printing enhances environmental sustainability by limiting waste and the requirement for additional resources [30,31].

**Figure 8:**
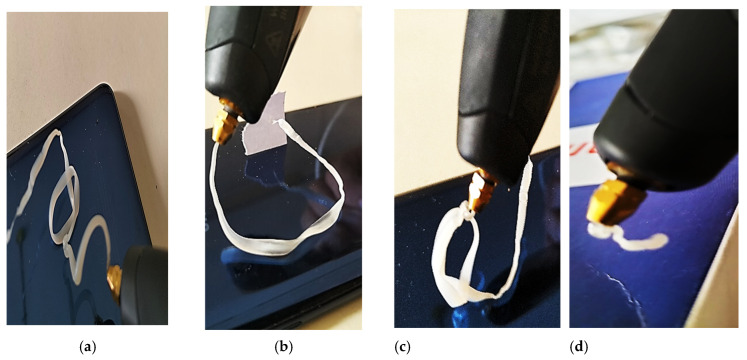
3D printing pen samples of (**a**) RPET filament, (**b**) RPET/GWNP_5%, (**c**) RPET/GWNP_10%, and (**d**) RPET/GWNP_15% composite filaments.

RPET and RPET/GWNP composite filaments were tested with 0.5 wt.% of coupling agent (according to optimized malic acid weight ratio obtained from Section 3.2.) using a 3D printing pen, as demonstrated in Figure 8. The results indicate that the RPET/GWNP_5% filament composite performed better in terms of shape, appearance, and stability than the RPET/GWNP_10% filament composite. However, increasing the GWNP ratio to 15% results in increased difficulty of printing with a pen as the filament begins to fracture. However, the RPET/GWNP_5% filament composite and RPET filament show comparable mechanical properties when printed using a 3D pen, stressing that a commitment to the environment demonstrated by recycling discarded glass must be displayed. This commitment allows for the creation of a new product with enhanced mechanical characteristics and reduced environmental impact. As a result, the RPET/GWNP_5% filament composite material appears to be an appropriate option for 3D printing.

## 4. Conclusions

This study contributes to the sustainable processing of waste materials by addressing multi-scale and multi-functional challenges, such as the reduction of heating energy required for glass trash upcycling, by implementing a straightforward and repeatable method. Additionally, the reduction of the accumulation of used drinking water bottles is facilitated by their recycling, which contributes to a variety of environmental issues. This can protect the ecosystem and offer a method of mitigating global warming. In order to generate eco-friendly and cost-effective 3D printing filaments, GWNP was utilized as a reinforcing agent in recycled drinking water bottle waste. The concentration of GWNP was varied from 5 wt.% to 15 wt.%, and malic acid was employed as a coupling agent. This study evaluated the performance of the ideal filament composite by conducting several chemical and physical analyses of these filaments. The results indicated that the thermal stability of the composites increased as the GWNP content was increased from 5 wt.% to 15 wt.%. Concurrently, the melt flow index declined with higher concentrations of GWNP and coupling agent. Furthermore, the tensile strength of the composites decreased in direct proportion to the increase in GWNP content. The RPET/GWNP_5% filament demonstrated satisfactory performance in 3D printing. The present study demonstrates the sustainable development of RPET when combined with other waste materials for 3D printing applications, as well as its potential for sustainable development. The versatility and capacity of this composite render it a valuable resource for innovative and eco-friendly sustainable 3D printing designs.

## Figures and Tables

**Table 1:** The central composite design (CCD) matrix and results for the two variables that influenced the tensile stress of the filament composite.

Trial	GWNP (A; wt.%)	Malic Acid (B; wt.%)	Response Tensile Stress (MPa)
1	15	1	20
2	10	1	42
3	5	1.5	48
4	15	0.5	24
5	5	1	61
6	10	1	42
7	10	1	42
8	10	1	42
9	10	1	42
10	10	0.5	56
11	5	0.5	51
12	10	1.5	18
13	15	1.5	16

**Table 2:** ANOVA analysis for response function of the tensile stress.

Source	Sum of Squares	df	Mean Square	F-Value	*p*-Value	
Model	2234.53	2	1117.27	30.71	<0.0001	significant
A-GWNP	1710.62	1	1710.62	47.02	<0.0001	
B-MA	523.91	1	523.91	14.40	0.0035	
Residual	363.78	10	36.38			
Lack of Fit	363.78	6	60.63			
Pure Error	0.0000	4	0.0000			
Cor Total	2598.31	12				
Std. Dev.	6.03		R^2^	0.860		
Mean	38.77		Adjusted R^2^	0.832		
C.V. %	15.56		Predicted R^2^	0.712		
			Adeq Precision	15.689		

## Data Availability

All data are included in the manuscript.
